# Participation in Older Adult Physical Activity Programs and Risk for Falls Requiring Medical Care, Washington State, 2005–2011

**DOI:** 10.5888/pcd12.140574

**Published:** 2015-06-11

**Authors:** Mikael Anne Greenwood-Hickman, Dori E. Rosenberg, Elizabeth A. Phelan, Annette L. Fitzpatrick

**Affiliations:** Author Affiliations: Dori E. Rosenberg, Group Health Research Institute, Seattle, Washington; Elizabeth A. Phelan, Department of Medicine, School of Public Health and Community Medicine, University of Washington, Seattle, Washington; Annette L. Fitzpatrick, University of Washington, Seattle, Washington. At the time of this study, Ms Greenwood-Hickman was affiliated with the University of Washington, Seattle, Washington, and Group Health Research Institute, Seattle, Washington.

## Abstract

**Introduction:**

Physical activity is known to prevent falls; however, use of widely available exercise programs for older adults, including EnhanceFitness and Silver Sneakers, has not been examined in relation to effects on falls among program participants. We aimed to determine whether participation in EnhanceFitness or Silver Sneakers is associated with a reduced risk of falls resulting in medical care.

**Methods:**

A retrospective cohort study examined a demographically representative sample from a Washington State integrated health system. Health plan members aged 65 or older, including 2,095 EnhanceFitness users, 13,576 Silver Sneakers users, and 55,127 nonusers from 2005 through 2011, were classified as consistent users (used a program ≥2 times in all years they were enrolled in the health plan during the study period); intermittent users (used a program ≥2 times in 1 or more years enrolled but not all years), or nonusers of EnhanceFitness or Silver Sneakers. The main outcome was measured as time-to-first-fall requiring inpatient or out-of-hospital medical treatment based on the *International Classification of Diseases, 9th Revision, Clinical Modification*, Sixth Edition and E-codes.

**Results:**

In fully adjusted Cox proportional hazards models, consistent (hazard ratio [HR], 0.74; 95% confidence interval [CI], 0.63–0.88) and intermittent (HR, 0.87; 95% CI, 0.8–0.94) EnhanceFitness participation were both associated with a reduced risk of falls resulting in medical care. Intermittent Silver Sneakers participation showed a reduced risk (HR, 0.93; 95% CI, 0.90–0.97).

**Conclusion:**

Participation in widely available community-based exercise programs geared toward older adults (but not specific to fall prevention) reduced the risk of medical falls. Structured programs that include balance and strength exercise, as EnhanceFitness does, may be effective in reducing fall risk.

## Introduction

Falls affect 30% to 40% of community-living adults over age 65 every year. Half of falls result in some type of injury, and falls may contribute to declining function, loss of independence, illness, and death (1). Fall-related health care costs the United States nearly $30 billion annually (2). For many older adults, fear of falls and their physical and psychological aftermath are a serious concern, often leading to self-imposed restrictions on activity (3,4).

Fall prevention research has found that regular physical activity incorporating strength and balance exercise can reduce falls, fall-related injuries, and falls resulting in medical care (5–7). Community-based programs intended to enhance older adults’ access to age-appropriate exercise may be beneficial in preventing falls. In a large integrated health system in Washington State, Group Health Cooperative (GHC), Medicare-qualifying adult members aged 65 or older are eligible to participate in 2 nationally disseminated exercise programs at no additional cost. EnhanceFitness (EF), an evidence-based intervention funded by the Centers for Disease Control and Prevention, offers community-based group exercise classes led by qualified instructors. Another program, Silver Sneakers (SS), provides full membership to participating fitness centers nationwide in more than 10,000 locations (8,9). Previous investigations have suggested that specific fall prevention exercise programs can be effective in preventing falls among community-dwelling older adults (10,11). However, these programs targeted fall prevention and were not as widely disseminated as EF and SS are. Although general exercise programs for older adults reach a much wider audience, their impact on fall prevention has not been studied. Although EF participation has been shown to improve physical function (12,13), no previous studies have examined associations between participation in either EF or SS and fall-related outcomes (14). Preventing falls and the costs and system burden they create is a relevant outcome from both the societal and health plan perspectives.

Our objective was to examine the relationship between participation in EF or SS and risk for a fall requiring medical treatment (termed “medical fall” hereafter), in a sample of GHC Medicare plan enrollees. On the basis of existing evidence that physical activity plays an important role in fall prevention, we hypothesized that consistent users of either program would have lower risks of a medical fall compared with nonusers.

## Methods

### Study design and sample

A retrospective cohort study was conducted among a demographically representative sample of the Seattle-area population of older adults. The exposure of interest was defined as participation in either the EF or SS program and the outcome was the first occurrence of a medical fall during the study period (2005–2011). Participants were members older than age 65 of GHC, which serves patients throughout the states of Washington and Northern Idaho. Participants were selected by using the following eligibility criteria: Integrated Group Practice members (receiving medical care primarily within the GHC system), continuous enrollment for at least 1 year, aged 65 to 98, and eligible for the Medicare EF and SS programs for some portion of 2005 through 2011. Individuals were excluded if they met any of the following specific criteria: residing in long-term care or nursing home setting, receiving hospice care (International Classification of Diseases, 9th Revision, Clinical Modification [ICD-9-CM] code V66.7), wheelchair-bound (V46 or V53.8), aged 99 or older, or having a diagnosis of a serious mental health or substance use disorder (290–319.99, not including depression [296.2, 296.3, 300.4, 311] anxiety [300.02], or dementia [290]). All demographic, health, and medical record data were extracted from GHC electronic health records and merged with participation data supplied by the EF and SS programs. GHC has complete records of health care use and costs, and these data have been validated and are frequently used for research (15). This research was approved by the institutional review boards of both Group Health Research Institute and the University of Washington.

EF, a nationally disseminated, evidence-based exercise program (16) for older adults, offers group-based exercise classes in community settings. Each class lasts 1 hour and follows a set format, including exercises targeting cardiovascular endurance (20–25 min), strength (20 min), and balance and flexibility (10 min), all of which are adaptable to individual ability level (8,17). SS is a benefit offered to Medicare Advantage enrollees that allows access to more than 10,000 fitness facilities nationwide. People who enroll in SS are granted access to exercise equipment and group exercise classes offered through selected fitness centers. SS also offers older adult fitness classes through these facilities that participants may choose to attend (9).

### Exposure and outcome assessments

Participation in either program was defined as documentation of attendance at either the EF or SS programs at least twice in a given year. Because specific attendance counts were not available in the data set, participation in the EF and SS programs was stratified into 3 levels in an effort to approximate regular exposure to the programs. These strata were 1) consistent users, who participated every year they were enrolled in GHC during the study period (2005–2011); 2) intermittent users, who participated at least 1 but not all years they were enrolled; and 3) nonusers, who never participated in either program while enrolled. Mean duration of enrollment in GHC for this sample was 4.7 (standard deviation, 2.3) years.

Falls requiring medical treatment were identified through the use of both inpatient and outpatient recording of ICD-9-CM codes (805–829: fractures, including hip fracture; 830–839: joint dislocations; and 800–804 and 850–854: intracranial injury) and E-codes (880–888: accidental fall injury) indicating medical treatment of an injury related to having had a fall (18). The presence of an E-code or one of the listed ICD-9-CM codes was sufficient to define a study outcome consistent with definitions developed in previous research (2,19).

### Covariates

The following covariates were extracted from electronic health record data of both inpatient and outpatient treatment in the GHC system and were assessed as potential confounders to the relationship of interest (ie, the relationship between program participation and risk of a medical fall): age (continuous), sex (male/female), race/ethnicity (white, black, Asian, Hispanic, Native American), body mass index (BMI, kg/m^2^, calculated from most recent height and weight for each year; continuous), smoking status (yes/no), and Charlson Comorbidity Index score (20), a general measure of comorbidity based on the presence or absence of 19 conditions weighted for severity (continuous). Small amounts of missing data were noted in the variables for race/ethnicity (6.1%), BMI (19.7%), and smoking (2.3%); models including these variables excluded individuals with missing data. Because of their strong association with increased fall risk, pharmacy data were used to indicate whether participants used sedatives or sleeping medications (2 fills within 90 days for benzodiazepines or prescription sleep medications; yes/no). 

The following comorbidities were identified through ICD-9 codes in the medical record and were also included in the analysis to account for their potentially confounding relationship with program participation and risk of a medical fall: diabetes status (249–251; yes/no); diagnosis of dementia (290, 294.1, 294.2, 331.0, 331.1, 331.82, 331.83; yes/no), walking disorder (719.7: difficulty walking; 781.2: abnormal gait; and 728.87: generalized weakness; yes/no), osteoarthritis (715, 721.0–721.9; yes/no), osteoporosis (733; yes/no), musculoskeletal conditions (712–719: arthropathy, rheumatoid arthritis, joint derangement; yes/no), and visual impairment (365: glaucoma; 366: cataract; 362.50–362.53, 362.55, 362.63: macular degeneration; and 362.01–362.03, 362.10, 362.11, 362.2, 363.31: retinopathy; yes/no). All comorbidity diagnosis variables were time-varying and constructed to reference a diagnosis for the given condition in the year before the year of interest.

### Data analysis

Initially, Kaplan–Meier survival curves were fit to the data to depict the time-to-medical-fall for each group. Time-to-event analyses were then conducted using days from entry into the study to the date of a fall, loss to follow-up, or the end of the study period (December 31, 2011). Individuals were censored if loss to follow-up occurred because of death or withdrawal from the GHC system. Specific dates for these events were unavailable and therefore defined as June 30 of the last year an individual appeared in the data set if that year was before 2011. A series of Cox Proportional Hazard models compared time-to-fall of nonusers to that of consistent and intermittent users of each program. Several models were constructed: a crude model (no adjustment for confounders), a demographic model (adjusted for age, race, and sex), and a full model (adjusted for age, race, sex, BMI, smoking, and the following comorbidities: dementia, walking disorder, osteoarthritis, osteoporosis, musculoskeletal conditions, and visual impairment). In fully adjusted models, individuals with missing values for a covariate were dropped from the analysis. All analyses were conducted using Stata 13 (StataCorp, LP).

## Results

Compared with nonusers, consistent and intermittent users of either program were more likely to be female, less likely to smoke, and had lower Charlson comorbidity scores (Tables 1 and 2). However, all users of either EF or SS were more likely to have had a diagnosis for osteoarthritis, osteoporosis, visual impairment, and musculoskeletal conditions during the study period, whereas consistent users of either EF or SS were less likely to use sedatives or sleeping medications compared with nonusers and intermittent users of either program. EF users had a mean attendance of 65 times in the year (median of 67), while SS users’ mean attendance was 51 times in 2011 (median of 33). Survival curves (Figure) suggest that consistent EF users had the greatest proportion of the sample remaining without a medical fall at the end of the study period, followed by intermittent EF users, and, finally, nonusers. Both consistent and intermittent SS groups had similar curves, with both showing reduced time to falls compared with the nonuser group.

**Table 1:** Demographic Characteristics of EnhanceFitness Users and Nonusers, Washington State, United States, 2005–2011

Trait^a^	Nonusers, n = 55,127	Consistent, n = 517	Intermittent, n = 1,578	*P* Value^b^
	n (%)			
**Mean age, y (range)**	74.1 (65–98)	73.7 (65–95)	75.0 (65–97)	<.001
**Female**	30,640 (55.6)	381 (73.7)	1,169 (74.1)	<.001
**Race^c^**				
White	45,471 (90.6)	436 (85.5)	1,356 (88.6)	<.001
Black	1418 (2.8)	21 (4.2)	60 (3.9)	
Asian	2,420 (4.8)	40 (7.9)	96 (6.3)	
Other	880 (1.8)	7 (1.4)	19 (1.2)	
**Mean BMI,^c^ kg/m^2^ (range)**	28.3 (7.4–77.1)	26.9 (16.3–45.7)	27.3 (14.9–49.4)	<.001
<18.5	531 (1.0)	4 (0.8)	12 (0.8)	<.001
18.5–24.9	8,851 (16.1)	142 (27.1)	367 (24.0)	
25.0–29.9	11,142 (20.2)	145 (28.1)	428 (27.1)	
>30.0	34,603 (62.8)	228 (44.1)	771 (48.9)	
**Smoker^c^**	4,359 (7.9)	22 (4.3)	59 (3.7)	<.001
**Charlson score^d^**	0.92 (0-18)	0.63 (0-10)	0.60 (0-10)	<.001
Diagnosis in study period				
Diabetes	10,984 (19.9)	87 (16.8)	278 (17.6)	<.001
Dementia	1,965 (3.6)	9 (1.7)	63 (4.0)	.002
Walking disorder^e^	624 (1.1)	9 (1.7)	37 (2.3)	.09
Osteoarthritis	22,801 (41.4)	277 (53.6)	990 (62.7)	<.001
Osteoporosis	873 (1.6)	22 (4.3)	63 (4.0)	<.001
Musculoskeletal condition^f^	27,254 (49.4)	330 (63.8)	1,151 (72.9)	<.001
Visual impairment^g^	31,894 (57.9)	371 (71.8)	1,271 (80.5)	<.001
Coronary heart disease	11,265 (20.4)	99 (19.2)	371 (23.5)	<.001
Hypertension	28,598 (51.9)	306 (59.2)	1,053 (66.7)	<.001
**Use of sedatives or sleeping medication**	7,106 (12.9)	43 (8.3)	208 (13.2)	<.001
**Fall resulting in medical treatment**	16,834 (30.5)	146 (28.2)	672 (42.6)	<.001

Abbreviation: BMI, body mass index; EF, EnhanceFitness.

a Unless otherwise specified, traits are described at first enrollment. Analysis of variance (for continuous covariates) comparing trends in the covariate across levels of either EF or SS participation.

b *P* values correspond to a Pearson’s χ^2^ analysis (for categorical covariates) or a 1-wayanalysis of variance (for continuous covariates) comparing trends in the covariate: race (6.1%), BMI (19.7%), and smoking (2.3%).

c The following variables have missing values for some individuals: race (6.1%), BMI (19.7%), and smoking (2.3%). Percentages are approximate.

d Charlson comorbidity score measures comorbidity based on the presence or absence of 19 conditions weighted for severity (continuous).

e Includes difficulty walking (719.7), abnormal gait (781.2), and generalized weakness (728.87).

f Includes arthropathy, rheumatoid arthritis, and joint derangement (712–719).

g Includes glaucoma (365), cataract (366), macular degeneration (362.50–362.53, 362.55, 362.63), and retinopathy (362.01–362.03, 362.10, 362.11, 362.2, 363.31).

**Table 2:** Demographic Characteristics of Silver Sneakers Users and Nonusers, Washington State, United States, 2005–2011

Trait^a^	Nonusers, n = 55,127	Consistent, n = 3,953	Intermittent, n = 9,623	*P* Value^b^
	n (%)			
**Mean age, y (range)**	74.1 (65–98)	70.0 (65–95)	71.5 (65–96)	<.001
**Female**	30,640 (55.6)	2,377 (60.1)	5,879 (61.1)	<.001
**Race^c^**				
White	45,471 (90.6)	3,462 (91.6)	8,571 (92.4)	<.001
Black	1,418 (2.8)	65 (1.7)	210 (2.3)	
Asian	2,420 (4.8)	202 (5.4)	388 (4.2)	
Other	880 (1.8)	49 (1.3)	109 (1.2)	
**Mean BMI,^c^ kg/m^2^ (range)**	28.3 (7.4–77.1)	27.9 (16.2–51.7)	28.6 (10.0–111.0)	<.001
<18.5	531 (1.0)	20 (0.5)	31 (0.3)	<.001
18.5–24.9	8,851 (16.1)	862 (21.8)	1,565 (16.3)	
25.0–29.9	11,142 (20.2)	1,145 (29.0)	2,253 (23.4)	
>30.0	34,603 (62.8)	1,926 (48.7)	5,774 (60.0)	
**Smoker^c^**	4,359 (7.9)	118 (3.0)	485 (5.0)	<.001
**Charlson score (range)^d^**	0.92 (0–18)	0.57 (0–10)	0.61 (0–10)	<.001
**Diagnosis in study period**				
Diabetes	10,984 (19.9)	572 (14.5)	1,968 (20.5)	<.001
Dementia	1,965 (3.6)	25 (0.6)	214 (2.2)	<.001
Walking disorder^e^	624 (1.1)	34 (0.9)	130 (1.4)	.09
Osteoarthritis	22,801 (41.4)	1,854 (46.9)	6,034 (62.7)	<.001
Osteoporosis	873 (1.6)	108 (2.7)	265 (2.8)	<.001
Musculoskeletal condition^f^	27,254 (49.4)	2,189 (55.4)	6,893 (71.6)	<.001
Visual impairment^g^	31,894 (57.9)	2,446 (61.9)	7,453 (77.5)	<.001
Coronary heart disease	11,265 (20.4)	561 (14.2)	2,226 (23.1)	<.001
Hypertension	28,598 (51.9)	1850 (46.8)	5,985 (62.2)	<.001
**Use of sedatives or sleeping medication**	7,106 (12.9)	344 (8.7)	1,357 (14.1)	<.001
**Fall resulting in medical treatment**	16,834 (30.5)	861 (21.8)	3,563 (37.0)	<.001

Abbreviation: BMI, body mass index; SS, Silver Sneakers.

a Unless otherwise specified, traits are described at first enrollment. Values are presented as n (%) unless otherwise indicated.

b *P* values correspond to a Pearson’s χ^2^ analysis (for categorical covariates) or a 1-way analysis of variance (for continuous covariates) comparing trends in the covariate across levels of program participation.

c The following variables have missing values for some individuals: race (6.1%), BMI (19.7%), and smoking (2.3%). Percentages are approximate.

d Charlson comorbidity score measures comorbidity based on the presence or absence of 19 conditions weighted for severity (continuous).

e Includes difficulty walking (719.7), abnormal gait (781.2), and generalized weakness (728.87).

f Includes arthropathy, rheumatoid arthritis, and joint derangement (712–719).

g Includes glaucoma (365), cataract (366), macular degeneration (362.50–362.53, 362.55, 362.63), and retinopathy (362.01–362.03, 362.10, 362.11, 362.2, 363.31).

**Figure:**
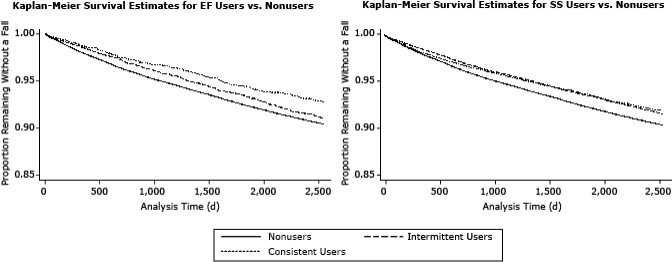
Kaplan–Meier survival curves of time to first medical fall for EnhanceFitness and Silver Sneakers users among enrollees of the Group Health Integrated Group Practice (Seattle, Washington), by consistent, intermittent, and nonusers.

In hierarchically adjusted Cox regression models (Table 3), a decreased risk of medical fall was found for both consistent and intermittent users of EF compared with nonusers, after adjusting for demographics. The same pattern remained in the fully adjusted model, showing consistent EF users to have a 26% decreased risk of a medical fall compared with nonusers, whereas intermittent EF users had a 13% decreased risk. The demographic model for SS use indicated a significant reduction for consistent and intermittent users. However, the full model yielded similar results for intermittent users (7% decrease in fall risk) but not for consistent SS users.

**Table 3:** Risk of a Medical Fall Among EF/SS Users Compared With Nonusers, Washington State, United States, 2005–2011

Group		N	Crude Model, HR (95% CI)	*P* Value	Demographic Model,^a^ HR (95% CI)	*P* Value	Full Model,^b^ HR (95% CI)	*P* Value
EF	Nonusers	55,127	1 [Reference]	—	1 [Reference]	—	1 [Reference]	—
	Intermittent	1,578	0.90 (0.84–0.98)	.009	0.84 (0.77–0.91)	<.001	0.87 (0.80–0.94)	.001
	Consistent	517	0.73 (0.62–0.86)	<.001	0.71 (0.60–0.84)	<.001	0.74 (0.63–0.88)	<.001

SS	Nonusers	55,127	1 [Reference]	—	1 [Reference]	—	1 [Reference]	—
	Intermittent	9,623	0.85 (0.82–0.88)	<.001	0.92 (0.88–0.95)	<.001	0.93 (0.90–0.97)	<.001
	Consistent	3,953	0.83 (0.78–0.89)	<.001	0.93 (0.87–0.99)	.03	0.95 (0.89–1.02)	.18

Abbreviations: CI, confidence interval; EF, EnhanceFitness; HR, hazard ratio; SS, Silver Sneakers..

a Model adjusted for age, sex, and race.

b Model adjusted for age, sex, race, and all covariates outlined in Tables 1 and 2.

## Discussion

To our knowledge, this is the first study to directly investigate the impact on the risk of medical falls of 2 exercise programs not specific to fall prevention. Consistent use of EF, our proxy for regular participation in the program over several years, was associated with the greatest reduction in risk of a medical fall, lowering risk by 20% to 30%. However, even intermittent use of the EF program also decreased the risk of medical falls. Although this finding corroborates previous evidence suggesting that strength and balance exercises, major components of the EF program, are essential to reducing fall and fall injury risk in older adults (5,21,22), further investigation involving more precise measure and categorization of participation is needed.

The results were less clear for the impact of the SS program. Unadjusted findings were suggestive of moderate risk reduction, but full adjustment yielded significant reduction only for intermittent users. This risk reduction was smaller than those seen for EF but was in the range of a 10% to 15% reduction in medical falls. However, the finding of significant impacts only for intermittent users runs counter to established findings in the literature, which suggest that consistent physical activity yields the strongest health impacts (5–7). This result makes drawing conclusions challenging and suggests that misclassification may be obscuring accurate interpretation of SS analyses.

Despite these limitations in the SS analysis, the magnitude of the association observed for SS participants was smaller than that seen with EF, a more structured program that includes balance and strength exercises that research suggests are critical to reducing fall risk (23). Because of the unstructured nature of the SS program in which participants can use whatever gym equipment or attend any classes they wish, we know very little about the type or intensity of exercise that participants engaged in or whether balance or strength exercises were performed. It is likely that balance exercises were not routinely practiced, as evidence suggests that most older adults do not engage in balance exercise (24,25). This likelihood may be part of the reason we did not see stronger associations between SS use and fall prevention.

EF has routinely scheduled classes multiple times per week and a strong social environment, promoting regular attendance at the EF program over many years. Users of the more free-form and independently driven SS program may be more likely to vary in the amount of use. On the basis of data supplied by each program, aggregate use statistics for this sample in 2011 support this differential attendance pattern. This finding suggests not only that EF users tend to use the program more frequently but also that the use pattern in the sample is more normally distributed (meaning that SS may have few very frequent users skewing the mean use statistic). This differential usage pattern may make the consistent and intermittent categories created for our analyses more problematic for SS, as the consistent category would be less likely to strongly parallel regular use. In short, although these results suggest that the EF program is more successful in reducing the risk of medical falls for this population, the nature of SS as an independently driven program with more sporadic attendance patterns lends itself more to misclassification under the participation definitions used in these analyses and makes conclusions about the program’s association with fall-related outcomes less robust.

This study has several limitations. First, it focuses only on falls resulting in medical care, as these appear in the inpatient or outpatient medical record, making it possible to measure without the use of self-report. This outcome definition excludes fall cases for which people do not seek medical attention and any injury truly due to a fall but not reported (and thus not coded) as such in the medical record. Despite this limitation, falls resulting in medical care are of high priority for risk reduction efforts given their adverse personal and societal effects (26,27).

Although missing data were not an issue for the outcome of interest, some data were missing for certain covariates, primarily race and BMI. Though these missing data resulted in small reductions in sample size for full adjustment models, they are not believed to have limited power. Participation in EF and SS is voluntary and, therefore, inherently self-selected. People who choose to participate in these programs may be systematically different from those who do not in ways that may affect their fall risk. Therefore, the potential for residual confounding remains. No information about engagement in physical activity outside either program was available for users or nonusers. The inability to adjust for baseline physical activity level in either group is a limitation; however, only 6% to 25% of older adults are estimated to regularly engage in the balance training and muscle-strengthening exercises requisite to fall risk reduction (28,29), suggesting that the impact of outside physical activity is likely low. Additionally, the threshold for participation in a given year was only 2 uses in that year, which is not indicative of regular physical activity through these programs. Although this finding limits our conclusions to the impact of 2 or more program uses per year, the low cut point used may have minimized the exposure of the group as a whole. This type of exposure misclassification would be expected to attenuate any association, rather than inflate it, so our results may be conservative. Future investigations should aim to use a continuous measure of participation to address this issue.

Despite these limitations, this study has several strengths. First, all outcomes and comorbidities were based on ICD-9-CM codes in the medical record rather than self-report. This procedure greatly reduces the potential for misclassification of comorbidity and outcome status, lending itself to a higher degree of accuracy in risk estimates. Furthermore, the use of this administrative data allowed for adjustment of many fall-related comorbidities that can be difficult to capture, including a history of gait and balance problems. Additionally, these analyses were based on a large, demographically representative sample, increasing power to detect associations and maximizing the generalizability of findings.

The results of this analysis provide evidence that participation in EF is associated with a reduced risk of medical falls. Furthermore, as hypothesized, this relationship shows a consistent pattern in which the strongest protective association was for consistent users of the program. Participation in SS may provide a moderate degree of fall protection, although findings were inconclusive. Overall, results suggest that evidence-based physical activity programs, particularly EF, should be more widely disseminated into communities not only for their general effects on fitness but also for their likely benefits on prevention of fall-related health care use, an important personal and societal outcome.
